# The Adomian Decomposition Method for Solving a Moving Boundary Problem Arising from the Diffusion of Oxygen in Absorbing Tissue

**DOI:** 10.1155/2014/579628

**Published:** 2014-08-04

**Authors:** Lazhar Bougoffa

**Affiliations:** Department of Mathematics, Faculty of Science, Al Imam Mohammad Ibn Saud Islamic University (IMSIU), P.O. Box 90950, Riyadh 11623, Saudi Arabia

## Abstract

This paper begins by giving the results obtained by the Crank-Gupta method and Gupta-Banik method for the oxygen diffusion problem in absorbing tissue, and then we propose a new resolution method for this problem by the Adomian decomposition method. An approximate analytical solution is obtained, which is demonstrated to be quite accurate by comparison with the numerical and approximate solutions obtained by Crank and Gupta. The study confirms the accuracy and efficiency of the algorithm for analytic approximate solutions of this problem.

## 1. Introduction

The solution of the oxygen diffusion problem in a medium [1], which simultaneously absorbs the oxygen, consists of finding *u* and *s* such that

(1) $$\begin{array}{c} \frac{\partial u}{\partial t}=\frac{\partial^{2}u}{\partial x^{2}}-1,\;0<x<s\left(t\right), \end{array}$$

subject to

(2) $$\begin{array}{c} \frac{\partial u}{\partial x}\left(t,0\right)=0, \end{array}$$

(3) $$\begin{array}{c} u\left(t,s\left(t\right)\right)=0, \end{array}$$

(4) $$\begin{array}{c} \frac{\partial u}{\partial x}\left(t,s\left(t\right)\right)=0 \end{array}$$

and the initial condition

(5) $$\begin{array}{c} u\left(0,x\right)=\frac{1}{2}{\left(1-x\right)}^{2},\;0<x<s\left(0\right)=1. \end{array}$$

The above equations represent a moving boundary problem, and since not only the concentration of oxygen is always zero at the boundary but also, in addition, no oxygen diffuses across the boundary at any time, there is no relationship which contains the velocity of the moving boundary explicitly.

On comparison of this problem with the one-phase Stefan problem, we observe that the Neumann boundary condition is different to the Stefan condition, which explicitly contains the velocity of the moving boundary [1, 2].

Problems such as this have been treated under the name of Crank and Gupta problem and approached by numerical solutions. This is an example of a nonlinear parabolic moving boundary problem, which is difficult to get the exact solution.

Many approximate methods have been used to solve this type of problems, for example, the numerical method [2–9] and the method applies the Keller box finite difference scheme [10–12].

Crank, Gupta and Banik [1–3] were the first to consider integral methods applied to the oxygen diffusion problem. The integral methods have been also discussed in [13].

In [1], the authors proposed the following polynomial profile of fourth degree centered at *x* = *s*, for the resolution of (1)–(5):

(6) u1(t,x)=s22(1−xs)2+(4u0(t)−s2)(1−xs)3 −(3u0(t)−s22)(1−xs)4,

where $u_{0}(t)=1/2-2\sqrt{t/\pi }$, and then obtained

(7) $$\begin{array}{c} \frac{d}{dt}\overset{s}{\underset{0}{\int }}u\;dx=-s, \end{array}$$

which leads to an ODE:

(8) $$\begin{array}{c} s^{\prime }\left(t\right)=\frac{\left(20+8u_{0}^{\prime }\left(t\right)\right)s}{8u_{0}\left(t\right)+s^{2}}\;\text{with}\;t\geq \frac{4}{25\pi }. \end{array}$$

A similar analysis has been applied in [2] for the following polynomial profile of fourth degree centered at *x* = 0:

(9) u2(t,x)=u0+12(s2−12u0)(xs)2 +(8u0−s2)(xs)3+12(s2−6u0)(xs)4,

where *u*_0_(*t*) = *u*(*t*, 0) and *s* is determined from

(10) $$\begin{array}{c} \frac{d}{dt}\overset{s}{\underset{0}{\int }}xu\;dx=u_{0}\left(t\right)-\frac{1}{2}s^{2}. \end{array}$$

The resulting ODEs are given by

(11) $$\begin{array}{c} \frac{du_{0}}{dt}=-\frac{5s^{4}+30s^{2}u_{0}+24u_{0}^{2}}{s^{2}\left(5s^{2}+24u_{0}\right)},\;\;\frac{ds}{dt}=-\frac{60\left(s^{2}-4u_{0}\right)}{s\left(5s^{2}+24u_{0}\right)}. \end{array}$$

The purpose of this paper is to apply the Adomian decomposition method [14–37] to find the solution of (1), (3), and (4), that is, the oxygen diffusion *u*(*t*, *x*), and then obtain an expression for the location of the moving boundary, which gives an ODE to solve for *s*(*t*) as a function of time. In addition, we will show that the partial solution in the *t*-directions requires less computational work by using the initial condition only. Also, using an a priori estimate, we prove the uniqueness of the solution of (1)–(5).

## 2. Analysis of the Method

Consider the general problem:

(12) $$\begin{array}{c} \frac{\partial u}{\partial t}=\frac{\partial^{2}u}{\partial x^{2}}-g\left(x\right),\;0<x<s\left(t\right), \end{array}$$

which is the governing equation, subject to the boundary condition

(13) $$\begin{array}{c} \frac{\partial u}{\partial x}\left(t,0\right)=h\left(t\right), \end{array}$$

the Dirichlet boundary condition

(14) $$\begin{array}{c} u\left(t,s\left(t\right)\right)=p\left(t\right), \end{array}$$

the Neumann boundary condition

(15) $$\begin{array}{c} \frac{\partial u}{\partial x}\left(t,s\left(t\right)\right)=q\left(t\right), \end{array}$$

and the initial condition

(16) $$\begin{array}{c} u\left(0,x\right)=\varphi \left(x\right),\;0<x<s\left(0\right)=1. \end{array}$$

Our problem contains, as a special case, the above system which describes the oxygen diffusion problem.

Based on the Adomian decomposition method, we write (12) in Adomian's operator-theoretic notation as

(17) $$\begin{array}{c} L_{xx}u=\frac{\partial u}{\partial t}+g\left(x\right), \end{array}$$

where

(18) $$\begin{array}{c} L_{xx}=\frac{\partial^{2}}{\partial x^{2}}. \end{array}$$

Applying the inverse linear operator *L*_*xx*_^−1^ = ∫_*x*_^*s*(*t*)^∫_*x*_^*s*(*t*)^(·)*dx* *dx* to (17) and taking into account that *u*(*t*, *s*(*t*)) = *p*(*t*) and (∂*u*/∂*x*)(*t*, *s*) = *q*(*t*), we obtain

(19) u(t,x)=p(t)−q(t)(s−x)+∫xs(t)∫xs(t)g(x)dx dx+∫xs(t)∫xs(t)∂u∂t dx dx.

Define the solution *u*(*t*, *x*) by an infinite series of components in the form

(20) $$\begin{array}{c} u\left(t,x\right)=\overset{\infty }{\underset{n=0}{\sum }}u_{n}\left(t,x\right). \end{array}$$

Consequently, the components *u*_*n*_ can be elegantly determined by setting the recursion scheme:

(21)  u0=p(t)−q(t)(s−x)+∫xs(t)∫xs(t)g(x)dx dx,un+1=∫xs(t)∫xs(t)∂un∂tdx dx, n≥0,

for the complete determination of these components.

Replace *p*(*t*) = *q*(*t*) = 0 and *g*(*x*) = 1 into the recursion scheme (21) to get

(22) u0=12!(s−x)2,u1=s′3!(s−x)3,u2=s′24!(s−x)4+s′′5!(s−x)5,  ⋯.

A polynomial profile of fifth degree is now obtained by the Adomian decomposition method, which is the truncated decomposition series *u*(*t*, *x*) = *u*_0_(*t*, *x*) + *u*_1_(*t*, *x*) + *u*_2_(*t*, *x*). So that

(23) $$\begin{array}{c} u\left(t,x\right)=\frac{1}{2!}{\left(s-x\right)}^{2}+\frac{s^{\prime }}{3!}{\left(s-x\right)}^{3}+\frac{s^{\prime 2}}{4!}{\left(s-x\right)}^{4}+\frac{s^{\prime \prime }}{5!}{\left(s-x\right)}^{5} \end{array}$$

and which automatically satisfies the boundary conditions (3) and (4).

We can now obtain an expression for the location of the moving boundary, *s*(*t*). This is derived from integrating (12) with respect to *x* from 0 to *x* and taking into account that (∂*u*/∂*x*)(*t*, 0) = *h*(*t*); we obtain

(24) $$\begin{array}{c} \frac{\partial u}{\partial x}=h\left(t\right)+\overset{x}{\underset{0}{\int }}g\left(x\right)dx+\overset{x}{\underset{0}{\int }}\frac{\partial u}{\partial t}dx. \end{array}$$

Substitute *x* = *s* into (24) and using the fact that (∂*u*/∂*x*)(*t*, *s*) = *q*(*t*). Thus

(25) $$\begin{array}{c} \overset{s(t)}{\underset{0}{\int }}g\left(x\right)dx+\overset{s(t)}{\underset{0}{\int }}\frac{\partial u}{\partial t}\;dx=q\left(t\right)-h\left(t\right),\;s\left(0\right)=1. \end{array}$$

Using the following Leibniz's rule for differentiation under the integral sign:

(26) $$\begin{array}{c} \frac{d}{dt}\overset{s(t)}{\underset{0}{\int }}u\left(t,x\right)dx=\overset{s(t)}{\underset{0}{\int }}\frac{\partial u}{\partial t}\;dx+u\left(t,s\left(t\right)\right)\frac{ds}{dt}, \end{array}$$

and taking into account that *u*(*t*, *s*(*t*)) = *p*(*t*), we obtain

(27) $$\begin{array}{c} \overset{s(t)}{\underset{0}{\int }}\frac{\partial u}{\partial t}\;dx=\frac{d}{dt}\overset{s(t)}{\underset{0}{\int }}u\left(t,x\right)dx-p\left(t\right)\frac{ds}{dt}. \end{array}$$

Substituting (27) into (25), we get

(28) $$\begin{array}{c} \overset{s(t)}{\underset{0}{\int }}g\left(x\right)dx+\frac{d}{dt}\overset{s(t)}{\underset{0}{\int }}u\left(t,x\right)dx-p\left(t\right)\frac{ds}{dt}=q\left(t\right)-h\left(t\right), \end{array}$$

where *s*(0) = 1. If we consider *p*(*t*) = *q*(*t*) = *h*(*t*) = 0 and *g*(*x*) = 1, then (28) becomes

(29) $$\begin{array}{c} \frac{d}{dt}\overset{s(t)}{\underset{0}{\int }}u\left(t,x\right)dx=-s. \end{array}$$

Substitute the profile equation (23) into (29) gives an ODE to solve for *s*(*t*), namely,

(30) $$\begin{array}{c} \frac{s^{2}s^{\prime }}{2!}+\frac{s^{3}s^{\prime 2}}{3!}+\frac{s^{4}s^{\prime \prime }}{4!}+\frac{s^{4}s^{\prime 3}}{4!}+3\frac{s^{5}s^{\prime }s^{\prime \prime }}{5!}+\frac{s^{6}s^{\prime \prime \prime }}{6!}=-s, \end{array}$$

with *s*(0) = 1. So that

(31) ss′2!+s2s′23!+s3s′′4!+s3s′34!+3s4s′s′′5!+s5s′′′6!+1=0.

We now can determine the location of the moving boundary *s*(*t*) as a function of time by solving the nonlinear equation (31). Indeed, the solution *s*(*t*) follows immediately by setting the following form:

(32) $$\begin{array}{c} s=\sqrt{1+2\lambda t}, \end{array}$$

where *λ* ∈ *R* is a parameter to be determined. Simple computations lead to

(33) $$\begin{array}{c} ss^{\prime }=\lambda ,\;\;s^{\prime \prime }s^{3}=-\lambda^{2},\\ s^{\prime \prime \prime }s^{5}=3\lambda^{3},\;\;s^{\prime \prime }s^{\prime }s^{4}=-\lambda^{3}. \end{array}$$

Substituting these expressions into (31), we obtain

(34) $$\begin{array}{c} \frac{\lambda }{2!}+\frac{\lambda^{2}}{3!}-\frac{\lambda^{2}}{4!}+\frac{\lambda^{3}}{4!}-3\frac{\lambda^{3}}{5!}+3\frac{\lambda^{3}}{6!}+1=0, \end{array}$$

or equivalently,

(35) $$\begin{array}{c} \lambda^{3}+6\lambda^{2}+24\lambda +48=0. \end{array}$$

Consequently, we find *λ* = −3.192143275966643 ≈ −3.2, which is a real root of this equation.

Hence, the concentration and the location of the moving boundary for 0 ≤ *t* < 1/6.4 ≈ 0.156 can be represented fairly accurately by the approximate expression equation (23) and

(36) $$\begin{array}{c} s=\sqrt{1-6.4t}, \end{array}$$

respectively.

It should be noted that this solution is applicable for the time 0 ≤ *t* < 1/6.4 only.

**Figure 1:**
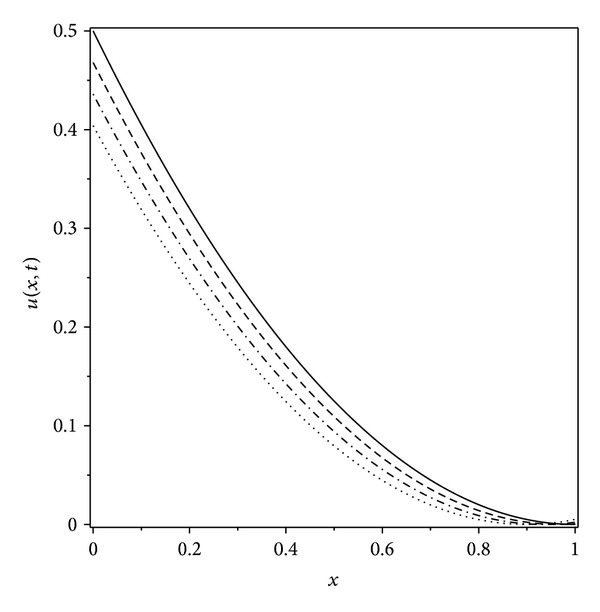
Concentration distributions *u*(*x*, *t*) for different values of *t*. Solid line: *t* = 0, dashed line: *t* = 0.01, dash dotted line: *t* = 0.02, and dotted line: *t* = 0.03.

**Figure 2:**
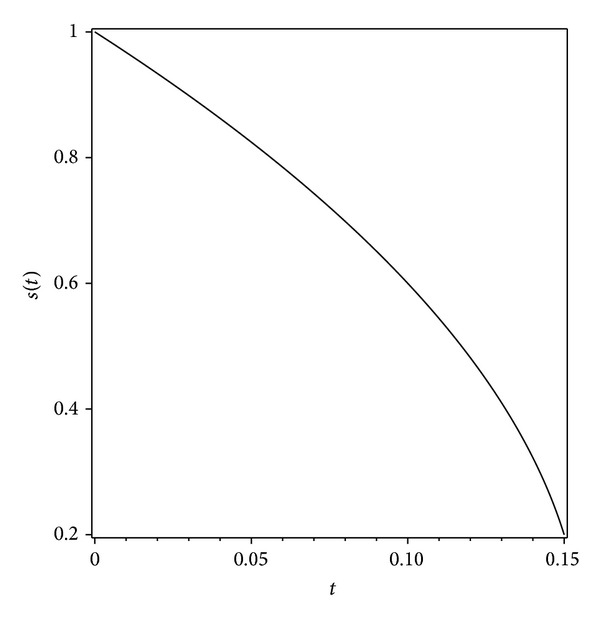
Variation of the moving boundary position equation (33) with the time *t* for 0 ≤ *t* < 0.156.

Graphs have been drawn to show the concentration distributions and the positions of the moving boundary at various times 0 ≤ *t* < 0.156 (Figures 1 and 2).

As it was mentioned in [13], we see that the method of Gupta and Banik [2] only starts at *t* = t* = 4/25*π*, with *s*(t*) = 1, and is applicable for the time interval 4/25*π* ≤ *t* ≤ *π*/16. For *t* ≥ 4/25*π*, Laplace solutions give analytical solutions for the short time problem [1]. Also, the Gupta and Banik method [2] requires that *s* ^2^ − 4*u*_0_ ≥ 0. Since *s*(0) = 1 it follows that *u*_0_(0) ≤ 0.25. This is incompatible with the initial condition at *t* = 0 and so this method can only start at *t* = t* > 0, with the assumption that *s*(t*) = 0.

Table 1 shows that the values obtained by using the Adomian decomposition method, which are in a very good agreement with those calculated by Gupta and Banik, for small times.

An expression for surface concentration can be obtained by putting *x* = 0 in (23). Thus

(37) $$\begin{array}{c} u\left(t,0\right)=\frac{1}{2!}s^{2}+\frac{s^{\prime }}{3!}s^{3}+\frac{s^{\prime 2}}{4!}s^{4}+\frac{s^{\prime \prime }}{5!}s^{5}, \end{array}$$

which can be compared with the numerical solutions [1]

(38) $$\begin{array}{c} u_{1}\left(t,0\right)=\frac{1}{2}-2\sqrt{\frac{t}{\pi }}. \end{array}$$

Comparative figures are given in Table 2.

An important note can be made here that the *t*-solution can be obtained by using the initial condition equation (16) only. To do this, we apply the inverse linear operator *L*_*t*_^−1^(·) = ∫_0_^*t*^(·)*dt* to both sides of (12) and use the initial condition equation (16) to obtain

(39) $$\begin{array}{c} u\left(t,x\right)=\varphi \left(x\right)-g\left(x\right)t+\overset{t}{\underset{0}{\int }}\frac{\partial^{2}u}{\partial x^{2}}\;dt, \end{array}$$

where *φ*(*x*) = (1/2)(1 − *x*)^2^ and *g*(*x*) = 1. So that the decomposition method consists of decomposing the unknown function *u*(*t*, *x*) into a sum of components defined by the series *u*(*t*, *x*) = ∑_*n*=0_^*∞*^ *u*_*n*_(*t*, *x*). Thus the components can be elegantly determined in a recursive manner as will be discussed later; we therefore set the recurrence scheme:

(40)   u0=12(1−x)2−t,un+1 =∫0t∂2un∂x2 dt, n≥0.

In view of this, the components *u*_0_(*t*, *x*), *u*_1_(*t*, *x*), *u*_2_(*t*, *x*),… are immediately determined as

(41)   u0=12(1−x)2−t,  u1=t,un+1=∫0t∂2un∂x2 dt=0, n≥1.

Consequently, the *t*-solution is readily found to be

(42) $$\begin{array}{c} u\left(t,x\right)=\frac{1}{2}{\left(1-x\right)}^{2}, \end{array}$$

which is a very good approximation and the same approximate solution obtained upon using the Laplace transforms when it has been assumed that the boundary has not moved from its original position, *s* = 1 [1].

To obtain *s*(*t*) as a function of time, substituting the profile equation (42) into (29), we get

(43) $$\begin{array}{c} \frac{ds}{dt}=-2\frac{s}{{\left(1-s\right)}^{2}},\;s\left(0\right)=1, \end{array}$$

which leads to the implicit solution

(44) $$\begin{array}{c} \ln s-2s+\frac{s^{2}}{2}=-2t-\frac{3}{2}. \end{array}$$

**Figure 3:**
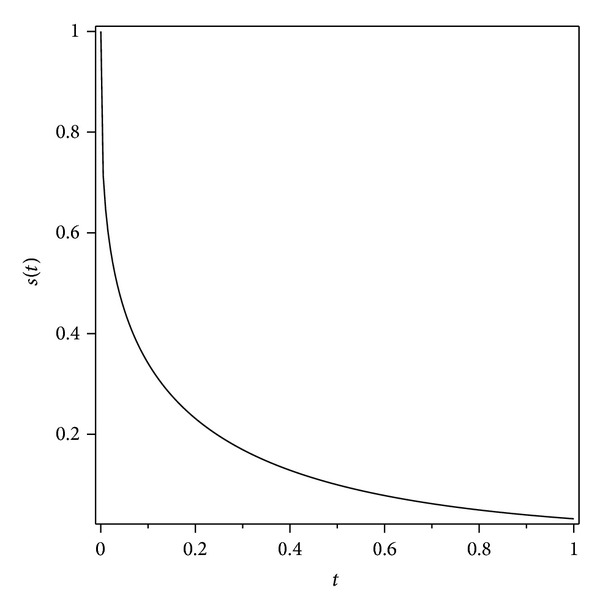
Moving boundary position *s*(*t*) in terms of *t* obtained from (44).

Graph has been drawn to show the solution for the moving boundary *s*(*t*) in Figure 3.

## 3. A Priori Estimate

Here we establish an a priori estimate which ensures the uniqueness of the solution of the given free boundary value problem.

**Proposition 1 .**

For any solution *u*(*t*, *x*) of (12) that satisfies (2)–(5) there exists a positive constant *C* independent on *u*(*t*, *x*) such that

(45) sup⁡0≤τ≤T[∫0s(τ)u2(τ,x)dt dx+∫0τ∫0s(τ)(∂u∂x)2dt dx] ≤C(∫01g2(x)dx+∫01φ2(x)dx),

where *g* ∈ *L*_2_[0, *s*].

**Proof**

Multiply both sides of (12) by *u*, integrating over *Ω* ^*τ*^ = [0, *τ*]×[0, *s*(*τ*)]. After applying integration by parts and taking into account that *u*(*t*, *s*) = 0, (∂*u*/∂*x*)(*t*, *s*) = 0 and (∂*u*/∂*x*)(*t*, 0) = 0, we obtain

(46) 12∫0s(τ)u2(τ,x)dt dx−12∫0s(τ)φ2(x)dx +∫0τ∫0s(τ)(∂u∂x)2dt dx=−∫0τ∫0s(τ)g(x)u(t,x)dt dx.

Use the *ϵ*-inequality: 2 | *ab* | ≤(1/*ϵ*)*a* ^2^ + *ϵb* ^2^, *ϵ* > 0 to estimate the term which arises in the right-hand side of (46).

Thus

(47) ∫0s(τ)u2(τ,x)dt dx+2∫0τ∫0s(τ)(∂u∂x)2dt dx ≤1ϵ∫0τ∫0s(τ)g2(x)dx+ϵ∫0τ∫0s(τ)u2(t,x)dt dx  +∫0s(τ)φ2(x)dx.

Since *Ω* ^*τ*^ = [0, *τ*]×[0, *s*(*τ*)]⊂[0, *T*]×[0,1], it follows

(48) ∫0s(τ)u2(τ,x)dt dx+∫0τ∫0s(τ)(∂u∂x)2dt dx ≤T∫01g2(x)dx+∫01φ2(x)dx  +ϵ∫0τ∫0s(τ)u2(t,x)dt dx.  

Using Gronwall's lemma, we obtain

(49) ∫0s(τ)u2(τ,x)dt dx+∫0τ∫0s(τ)(∂u∂x)2dt dx ≤C(∫01g2(x)dx+∫01φ2(x)dx).  

Now, replacing the right-hand side of (49) by its upper bound with respect *τ* in the interval [0, *T*], we obtain the desired inequality.

## 4. Conclusion

In this work we investigated the moving boundary problem arising from the diffusion of oxygen in absorbing tissue. The approximate method obtained upon using the ADM would specially be useful to calculate the concentration and the position of the moving boundary at an arbitrary time. Graphs have been drawn to show the concentration-distributions and the progress of the moving boundary with respect to time at various times. The work confirmed the power of the Adomian method in handling this example of a nonlinear parabolic moving boundary problem, without an exact solution.

## Figures and Tables

**Table 1:** A comparison for the position of the moving boundary as obtained from (a) numerical evaluation by using Runge-Kutta method, (b) approximation solution [1], (c) numerical method [1], and (d) ADM.

Values of *x*	Numerical solution	Approximate solution	Numerical method	ADM
0	—	—	—	1
0.051	1	1	0.9967	0.82073
0.060	0.9974	0.9996	0.9922	0.7848
0.080	0.9750	0.9817	0.9719	0.6985
0.100	0.9321	0.9393	0.9352	0.6000

**Table 2:** A comparison for the surface concentration *u*(*t*, 0) with *u*_1_(*t*, 0) [1].

Values of *x*	Analytical	Numerical	Approximate	ADM
0.04	0.274328	0.274496	0.274324	0.229152
0.08	0.180852	0.180969	0.180846	0.150304
0.12	0.109134	0.109228	0.109118	0.0714560
0.15	0.048771	0.048893	0.048648	0.0123200
